# Draft Genome Sequences of Two *Streptomyces* Isolates Obtained from Volcanic Soils in the Philippines

**DOI:** 10.1128/mra.01087-21

**Published:** 2022-02-10

**Authors:** Rey Vladimir Marasigan, Edwin Alcantara, Elcid Aaron Pangilinan, Francis Tablizo, El King Morado, Shiela Mae Araiza, Kris Punayan, Benedict Maralit, Ma. Anita Bautista

**Affiliations:** a Functional Genomics Laboratory, National Institute of Molecular Biology and Biotechnology, University of the Philippines Diliman, Quezon City, Philippines; b Core Facility for Bioinformatics, Philippine Genome Center, University of the Philippines Diliman, Quezon City, Philippines; c DNA Sequencing Core Facility, Philippine Genome Center, University of the Philippines Diliman, Quezon City, Philippines; d Microbial Insecticides Laboratory, Biotechnology for Natural Products Program, National Institute of Molecular Biology and Biotechnology (BIOTECH), University of the Philippines, Los Baños, Laguna, Philippines; SIPBS, University of Strathclyde

## Abstract

We report here the draft genome sequences of two *Streptomyces* isolates, namely, UNOB3_S3 and UNOC14_S4, obtained from volcanic soils in Albay, Philippines. The genome assemblies comprised 7.63 Mb and 8.24 Mb, respectively. Genome mining revealed genes and biosynthetic gene clusters that encode putative insecticidal products.

## ANNOUNCEMENT

*Streptomyces* produces secondary metabolites encoded by a group of genes known as biosynthetic gene clusters (BGCs). Some of these bioactive metabolites could be important sources of environmentally friendly insecticides (1). Existing classes of insecticides pose adverse effects to the environment and health of humans (2), prompting the development of safer but effective insecticides sourced from bacteria like *Streptomyces*. This study presents the assemblies and annotation of the genome sequences of 2 *Streptomyces* isolates for the subsequent mining of BGCs.

Isolates UNOB3_S3 and UNOC14_S4 were obtained from volcanic soils in Uno, Malilipot, Albay, Philippines using the serial dilution method (3). Colonies were isolated from 1 g of soil mixed and serially diluted with sterile distilled water. Aliquots (100 μL) from 10^−3^ and 10^−4^ dilutions were plated separately onto yeast malt agar (YMA; pH 7.0) consisting of the following: 4 g/L yeast extract, 4 g/L dextrose, 10 g/L malt extract, and 20 g/L agar. Colonies were picked after 5 days of incubation at 28°C.

Isolates UNOB3_S3 and UNOC14_S4 maintained at 4°C in YMA slants were cultivated for 3 days with continuous shaking (250 rpm at room temperature [RT]) in 50 mL yeast malt broth (YMB; pH 7.0), consisting of the following: 4 g/L yeast extract, 4 g/L dextrose, and 10 g/L malt extract. The final fermentation was performed in 450 mL YMB (pH 7.0) for 5 days with continuous shaking (250 rpm at RT). Bacterial cells pelleted by centrifugation (15 min and 12,000 × *g*) were subjected to genomic DNA extraction using the Zymo fungal/bacterial DNA mini prep DNA kit. Libraries were prepared using the Illumina Nextera DNA XT library preparation kit and sequenced using the MiSeq sequencing kit v3, 600 cycles (2 × 301 bp), on the Illumina MiSeq system RUO.

Sequencing generated read pairs of 1,043,574 and 652,426 for UNOB3_S3 and UNOC14_S4, respectively. The reads were trimmed using BBDuk from BBMap 38.86 with parameters ktrim=r, ref=adapters.fa, k=23, mink=11, hdist=1, trimq=15, qtrim=r, and minlen=30 (4). Error correction and assembly were done using SPAdes 3.15.3 with parameters --careful and --cov-cutoff auto (5). Contigs less than 500 bp long were discarded. The final assemblies were evaluated by BUSCO 5.1.3 (6) to assess completeness and by QUAST 5.1.0rc1 (7) to calculate the metrics of the assemblies.

The final assemblies for UNOB3_S3 and UNOC14_S4 consisted of 7.62 Mb and 8.24 Mb, 792 and 1,076 contigs, and GC contents of 71.98% and 71.44%, respectively. The BUSCO analysis against the streptomyces_odb10 (2020-03-06) database showed 96.9% and 97.1% completeness for UNOB3_S3 and UNOC14_S4, respectively (Table 1).

**TABLE 1:** Assessment of the assemblies of *Streptomyces* isolates UNOB3_S3 and UNOC14_S4

Parameter	Data by isolate	
	UNOB3_S3	UNOC14_S4
Genome size (bp)	7,618,150	8,235,973
No. of contigs	792	1,076
Largest contig (bp)	72,464	67,296
Coverage (×)	52	29
*N*_50_ (bp)	17,091	12,946
*L*_50_	143	190
Avg contig size	9,618.88	7,654.25
Total no. of genes	7,097	7,545
GC content (%)	71.98	71.44
BUSCO analysis (*n* = 1,579)		
Complete (%)	1,530 (96.9)	1,534 (97.1)
Complete and single copy (%)	1,525 (96.6)	1,526 (96.6)
Complete and duplicated (%)	5 (0.3)	8 (0.5)
Fragmented (%)	33 (2.1)	29 (1.8)
Missing (%)	16 (1)	16 (1)
GenBank accession no.	JAILXP000000000	JAINFC000000000
SRA accession no.	SRR15561083	SRR15561183

A phylogenetic analysis was done by aligning complete 16S rRNA gene sequences from the assemblies against the NCBI nonredundant/nucleotide (nr/nt) database. Streptomyces luteoverticillatus CGMCC 15060 (accession number GCA_003970715.1) was the closest to UNOB_S3, while Streptomyces olivoreticuli subsp. *olivoreticuli* ATCC 31159 (accession number GCA_003391135.1) was closest to UNOC14_S4, with 99.28% and 98.8% similarity between their 16S rRNA genes, respectively. A subsequent average nucleotide identity based on BLAST (ANIb) analysis using the Kostas Lab ANI/amino acid identity (AAI) matrix (8) reveals a 93.1% ANI between UNOB3_S3 and *S. luteoverticillatus* and 87.2% ANI between UNOC14_S4 and *S. olivoreticuli*.

Annotation using NCBI PGAP version 2021-07-01.build5508 (9) predicted 7,097 genes for UNOB3_S3 and 7,545 genes for UNOC14_S4. Notable were GH18 chitinases, of which members have been shown to be insecticidal (10). antiSMASH version 6.0.1 (11) predicted regions containing BGCs, namely, 77 regions for UNOB3_S3 and 85 for UNOC14_S4. Studies are under way to confirm and elucidate their roles.

### Data availability.

The genome sequences of UNOB3_S3 and UNOC14_S4 have been deposited in DDBJ/ENA/GenBank under the accession numbers JAILXP000000000 and JAINFC000000000, respectively. The versions described in this paper are the first versions, JAILXP010000000 and JAINFC010000000. Their respective SRA accession numbers are SRR15561083 and SRR15561183.
